# Similar to Spironolactone, Oxymatrine Is Protective in Aldosterone-Induced Cardiomyocyte Injury via Inhibition of Calpain and Apoptosis-Inducing Factor Signaling

**DOI:** 10.1371/journal.pone.0088856

**Published:** 2014-02-13

**Authors:** Ting-Ting Xiao, Yuan-Yuan Wang, Yan Zhang, Cong-Hui Bai, Xiang-Chun Shen

**Affiliations:** 1 Research Division of Pharmacology, Guiyang Medical University, Guiyang, China; 2 School of Chinese Medicine, Hong Kong Baptist University, Kowloon Tang, Kowloon, Hong Kong, China; 3 Research Division of Pathophysiology, Guiyang Medical University, Guiyang, China; 4 School of Life Science,Zhejiang Sci-Tech University, Hangzhou, China; The University of Texas MD Anderson Cancer Center, United States of America

## Abstract

Accumulating evidence indicates that oxymatrine (OMT) possesses variously pharmacological properties, especially on the cardiovascular system. We previously demonstrated that activated calpain/apoptosis-inducing factor (AIF)-mediated pathway was the key molecular mechanism in aldosterone (ALD) induces cardiomyocytes apoptosis. In the present study, we extended the experimentation by investigating the effect of OMT on cardiomyocytes exposed to ALD, as compared to spironolactone (Spiro), a classical ALD receptor antagonist. Cardiomyocytes were pre-incubated with OMT, Spiro or vehicle for 1 h, and then, cardiomyocytes were exposed to ALD 24 h. The cell injury was evaluated by 3-(4,5-dimethylthiazol-2-yl)-2,5-diphenyltetrazolium bromide (MTT) assay and lactate dehydrogenase (LDH) leakage ratio. Apoptosis was determined by terminal deoxynucleotidyl transferase-mediated dUTP nick-end labeling (TUNEL) assay, annexin V/PI staining, and relative caspase-3 activity assay. Furthermore, expression of pro-apoptotic proteins including truncated Bid (tBid), calpain and AIF were evaluated by western blot analysis. ALD stimulation increased cardiomyocytes apoptosis, caspase-3 activity and protein expression of calpain, tBid and AIF in the cytosol (p<0.05). Pre-incubated with cardiomyocytes injury and increased caspase-3 activity were significantly attenuated (p<0.05). Furthermore, OMT suppressed ALD-induced high expression of calpain and AIF. And these effects of OMT could be comparable to Spiro. These findings indicated that OMT might be a potential cardioprotective-agent against excessive ALD-induced cardiotoxicity, at least in part, mediated through inhibition of calpain/AIF signaling.

## Introduction

Nearly 40% of deaths are related to cardiovascular disease in developed countries [1]. Heart failure, characterized by impaired systolic and/or diastolic function with high morbidity and mortality, is the common ending of diverse etiologies. It is well accepted that one of most important reasons resulting in heart failure is cardiomyocytes loss including necrosis and apoptosis. The apoptotic process involves many factors, such as the sympathetic nervous system, renin-angiotensin system, reactive oxygen species, etc. Increasing evidence indicates that excessive aldosterone (ALD) may play a critical role in the process of heart failure, and heart is one of direct targets of ALD, which can provoke hypertrophy and apoptosis of cardiomyocytes [2]. ALD could induce cardiomyocytes apoptosis via [Ca^2+^]_i_ overload-mediated mitochondria-dependent and independent pathway [3], [4], [5], [6]. Accumulating experimental data indicates that calcium/calpain-dependent mechanism was involved in the ALD-induced renal cell apoptosis [7], [8] and myocardial apoptosis [9], [10]. Moreover, the activation of calpain and apotosis-inducing factor (AIF) signaling involvement in ALD-mediated cardiomyoctes apoptosis has been demonstrated by inhibition of Ca^2+^ chelator and calpain inhibitor [10]. It is well accepted that spironolactone (Spiro) protects against heart failure by inhibiting myocardial apoptosis and fibrosis *in vivo* and *in vitro* [9], [11], [12]. Nowadays, Spiro is the first choice in clinic practice for the patients who suffer from heart failure accompany with high plasma concentration of ALD [13], [14], [15].

Oxymatrine (OMT) (C_15_H_24_N_2_O) is the main active principle of Kushen (Sophorae flavescentis Radix), traditional Chinese herbal medicine made from the dried root of *Sophora flavescens* Ait. It has been demonstrated that OMT has wide-ranging of pharmacological effects, such as anti-inflammation, immune regulation, anti-virus, protection against acute lung injury and anti-hepatic fibrosis, etc. [16]. Abundant evidences show that OMT could prevent myocardium from apoptosis and fibrosis caused by lots of stimuli [17], [18], [19], [20], [21]. Hence, the aim of present study was to investigate the protective effect of OMT on ALD-induced apoptosis in cardiomyocytes. To the best of our knowledge, this is the first demonstration about the protective effect of OMT against ALD-mediated cardiomyocytes injury.

## Materials and Methods

### Chemicals and Animals

All chemicals and reagents were purchased from Sigma–Aldrich (St. Louis, MO, USA), unless stated otherwise. Between 1 and 3 days old Sprague-Dawley (SD) rats were provided by the experimental animal center of Guiyang Medical University. All animal procedures and experiments were performed following the approval of the Bioethics Committee of Guiyang Medical University.

### Cell Culture and Treatments

Primary cultured new born rats’ cardiomyocytes were prepared from SD rats between 1 and 3 days old according to previous methods [10]. The purity of cultured cardiomycoyte was >97% as evaluated by immunocytochemical staining using cardiac muscle sarcomeric α-actinin antibody (Boster Biotechnology, China). Cardiomyocyte treated with 0.2% dimethylsulfoxide (DMSO) (Amresco, USA) was served as a vehicle, and for other groups, treatment of either 25 µg/mL OMT (Green Valley Pharmaceutical Co. Ltd., Shanghai, China, with a purity of 98%), or 10 µM Spiro (National Institute for the control of pharmaceutical and biological products, China, purity >98%). After 1 h incubation, all medium was discarded and cells were treated with 10 µM ALD (Fluka, Switzerland, with a purity of 98%) for 24 h. All drugs were freshly dissolved in DMSO, and there was not significant effect on cardiomyocytes (data not shown).

### 3-(4,5-dimethylthiazol-2-yl)-2,5-diphenyltetrazolium Bromide (MTT) Assay

At the designated experimental time point, the supernatant of each well was removed, and 20 µL of 5 mg/mL MTT (Amresco, USA) solution was added into and incubated at 37°C for 4 h. Formazan salt crystals were then dissolved with 150 µL dimethylsulfoxide for each well. The mixtures are determined at 570 nm using a microplate reader (ELX800, GE, USA).

### Lactate Dehydrogenase (LDH) Leakage Ratio

LDH activity was measured using the relative LDH activity assay kit (KeyGEN, Nanjing, China). LDH leakage rate was expressed as the percentage of the total LDH activity (the extracellular LDH activity plus the intracellular LDH activity), according to the following equation: % LDH release rate = (LDH activity in medium/total LDH activity) ×100.

### Terminal Deoxynucleotidyl Transferase-mediated dUTP Nick-end Labeling (TUNEL) Assay

TUNEL (KeyGEN, Nanjing, China) assays were performed in accordance with the manufacturer’s protocol. Apoptotic cells were stained brown and normal cells were stained purple-blue. The percentage of TUNEL-positive cells was determined by counting at least 200 cells in 5 randomly selected fields.

### Annexin-V/PI Staining

The annexin-V-fluorescein isothiocyanate (FITC) apoptosis detection kit (KeyGEN, Nanjing, China) was used according to the manufacturer’s instruction. Observation through a fluorescence microscope (BX.51, OLYPUS, Japan), normal cells could only give off a weak green fluorescence. In the early stages of apoptosis, the cell membranes were stained with annexin V and gave off a vibrant green fluorescence, while the nucleolus was not stained with PI. The cells in the media and at late stages of apoptosis were highly stained by both annexin V and PI, therefore the cell membranes gave off green fluorescence and the nucleolus gave off a red fluorescence. The apoptotic cell counts were expressed as a percentage of the total number of cells giving off fluorescence.

### Caspase-3 Activity

The caspase-3 colorimetric assay kit (KeyGEN, Nanjing, China) was used to determine caspase-3 activity following the instruction of the manufacturer’s protocol. Cardiomycytes lysates were incubated with 1 M DTT and the labeled caspase-3 substrate DEVD-*p*-nitroanilide (DEVD-pNA) for 4 h at 37°C. Cleavage of a substrate was quantified by measuring the absorbance at 405 nm using a microplate reader (ELX800, GE, USA).

### Western Blot Analysis

The primary cultured cells were washed once in PBS and lysed on ice in lysis buffer (Beyotime, Jiangsu, China) after treatments were completed. The protein concentration was determined using a BCA protein assay kit (Beyotime, Jiangsu, China). Equal amounts of proteins were subjected to 8–12% SDS-polyacrylamide gel. After being electrophoresed, the proteins were transferred onto a PVDF membrane by using a Bio-Rad western blot analysis apparatus. The membrane was incubated with blocking buffer for 1.5 h at room temperature and then incubated overnight at 4°C with the primary polyclonal antibodies against calpain, tBid and AIF (1∶500, Santa Cruz Biotechnology, Santa Cruz, CA, USA), followed by incubation with corresponding secondary antibodies. Specific protein bands were visualized with an ECL advanced western blot analysis detection kit (Beyotime, Jiangsu, China).

### Statistical Analysis

Data were expressed as mean ± standard error of the mean (S.E.M.) of at least 3 independent experiments. Between-group comparisons were performed by using t-test, statistical significance was set at P<0.05, P<0.01 was considered extraordinarily significant.

## Results

### OMT Protected Cardiomycoytes from ALD-induced Cell Injury

**Figure 1:**
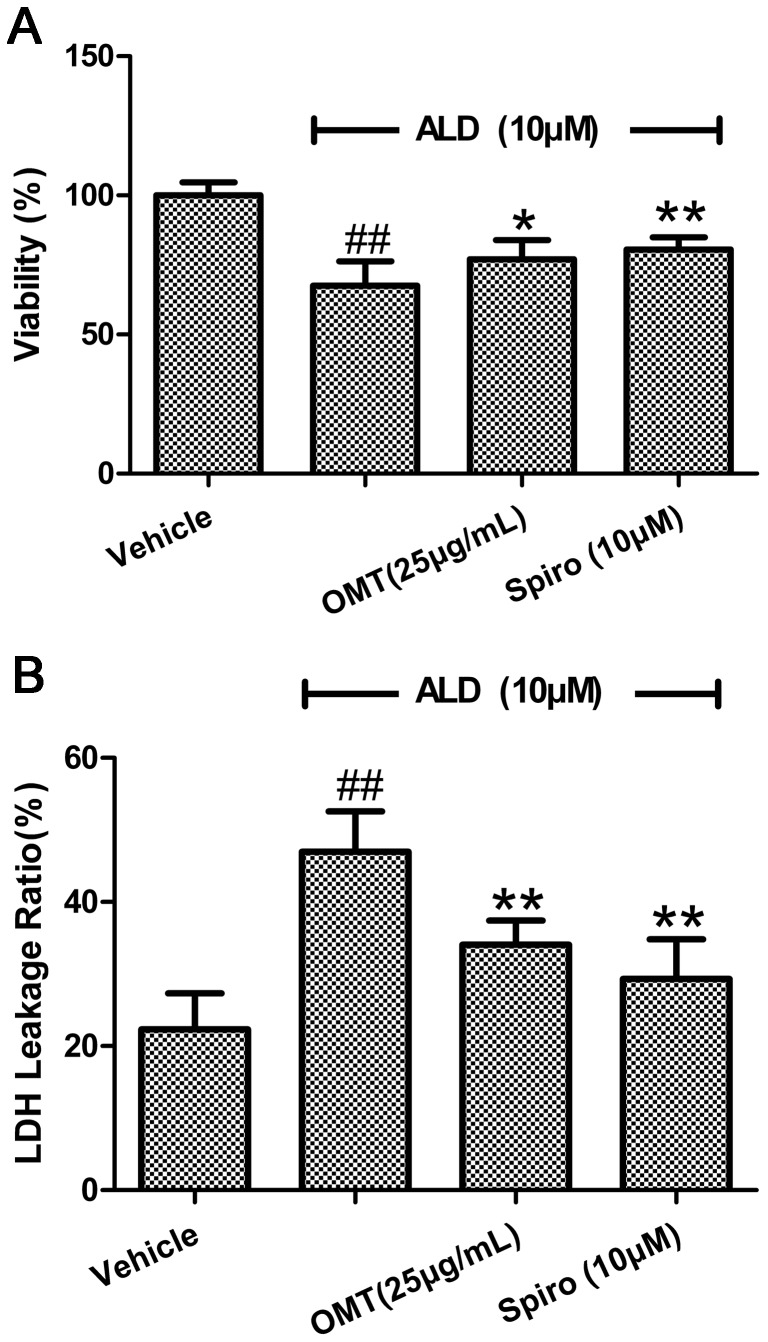
Inhibited effects of OMT on ALD-induced cardiomyocytes viability and LDH release. After an equal number of cardiomyocytes were pre-treated with either Spiro (10 µM), or OMT (25 µg/mL) for 1 h and then were stimulated with ALD (10 µM) for 24 h. At the end of the incubation period, (A) the cells were evaluated for viability with MTT; (B) cell death was measured by an LDH release ratio assay. The results (mean ± SEM) are from 6 series of experiments, each carried out in triplicate.^ ##^P<0.01 compared with vehicle. *P<0.05 compared with ALD. **P<0.01 compared with ALD.

Previous study showed that ALD caused cardiomyocytes injury was in the time- and dose-dependent manners and at the dose of 10 µM for 24 h caused 33.33% cells death in MTT assay [10]. At present study, the MTT assay showed that cardiomyocyte viability was increased from 67.61% to 77.09% by being pre-treated with 25 µg/mL OMT 1 h, the cell damage was also attenuated by Spiro pre-treatment (Figure 1(A)). LDH leakage ratio is served as a biomaker of cellular membrane injury. There was a significant increase in LDH leakage ratio (P<0.01 compared with the vehicle group) in cells exposed to 10 µM ALD alone for 24 h. However, the high level of LDH leakage ratio was significantly reduced in cells pre-treated with OMT, as well as with Spiro (Figure 1(B)), and there was no difference between two pre-treated groups (P>0.05).

### OMT Protected Against Cardiomyocytes from ALD-induced Cell Apoptosis

**Figure 2:**
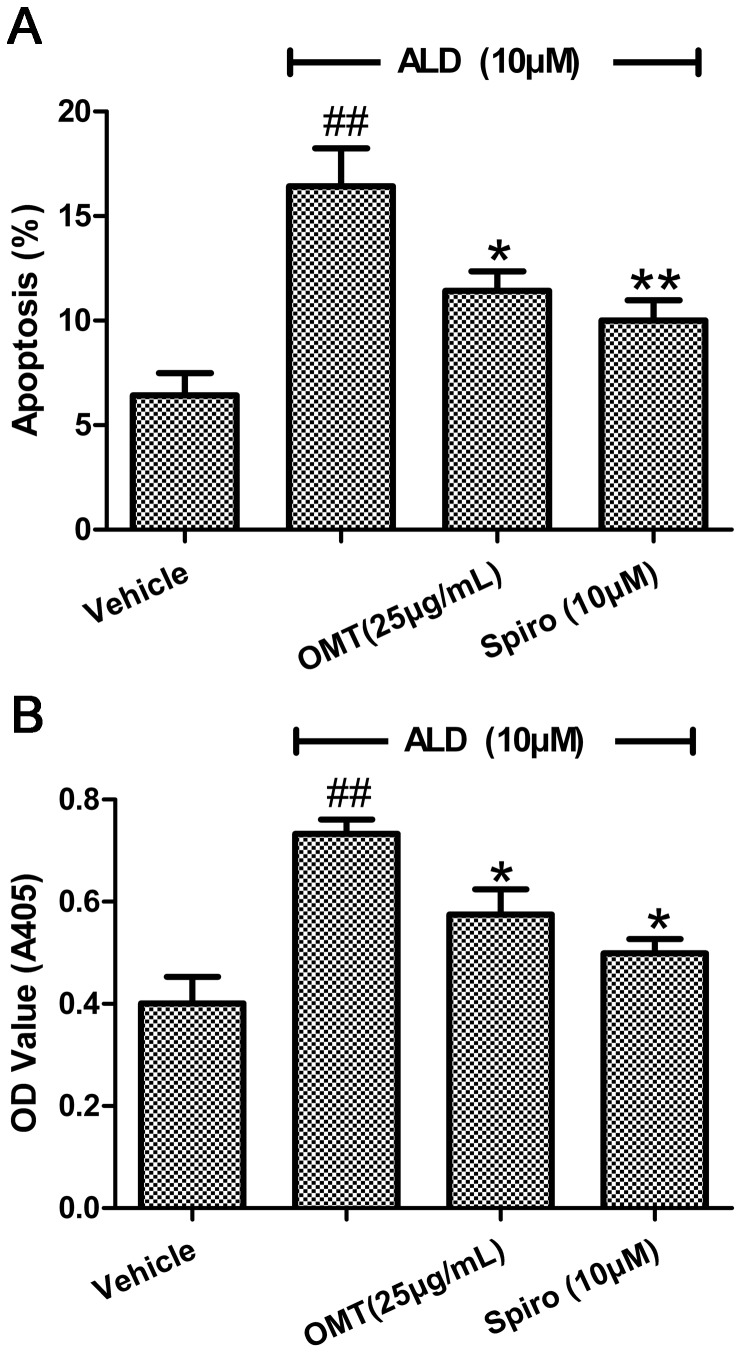
Impacts of OMT on ALD-induced cardiomyocytes apoptosis by annexin V/PI staining and caspase-3 activity analysis. Cardiomyocytes were pre-treated with either Spiro (10 µM), or OMT (25 µg/mL) for 1 h and then were stimulated with ALD (10 µM) for 24 h. At the end of the incubation period, the myocytes were then prepared for annexin V/PI staining. (A) The data expressed as % apoptotic cardiac myocytes. (B) The cells were evaluated for caspase-3 activity. The results (mean ± SEM) are from 6 series of experiments, each carried out in triplicate.^ ##^P<0.01 compared with vehicle. *P<0.05 compared with ALD. **P<0.01 compared with ALD.

The apoptotic cells were observed and calculated after annexin-V/PI staining. The pro-apoptotic impact of ALD on cardiomyocytes is obvious (P<0.01 compared to the vehicle group). The percentage of apoptotic cells reached to 16.43% in the ALD alone treatment group, but it was remarkably reduced to 11.43% in the group pre-treated with OMT and to 10.00% in the Spiro pre-treatment group (Figure 2(A)).

The activated caspase-3 is a key executioner in apoptotic pathological process. The level of caspase-3 activity in the cells exposed to ALD alone was significantly higher than that in the vehicle group (P<0.01). However, when pre-incubation with OMT, the activation of caspase-3 was reduced from (0.73±0.06) to (0.58±0.10) (P<0.05 compared with the ALD group). The caspase-3 activity was decreased to (0.50±0.06) in the Spiro pre-treatment group (P<0.05 compared with the ALD group) (Figure 2(B)).

**Figure 3:**
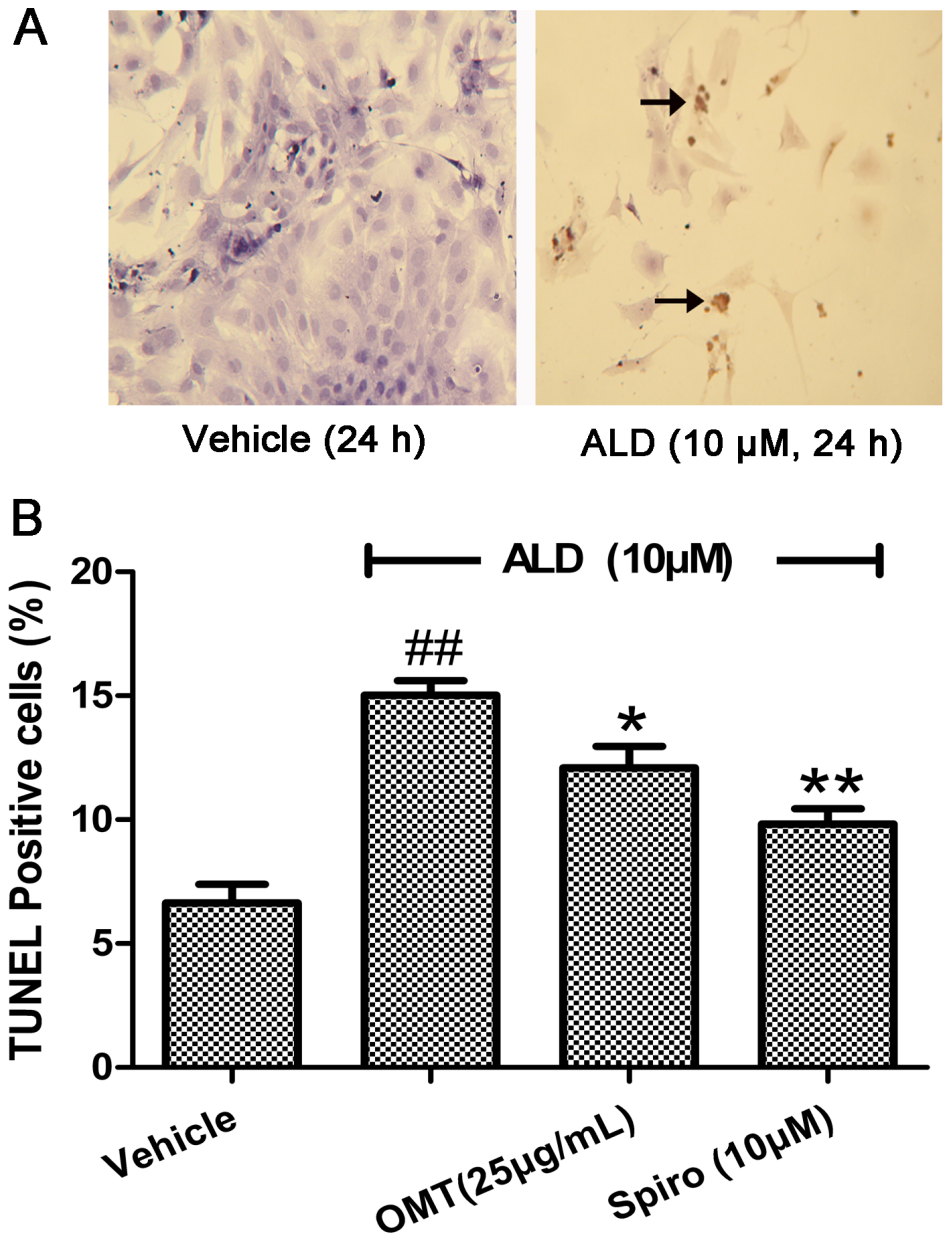
Protective effects of OMT on ALD-induced cardiomyocytes DNA fragmentation. Cardiomyocytes were pre-treated with either Spiro (10 µM), or OMT (25 µg/mL) for 1 h and then were stimulated with ALD (10 µM) for 24 h. At the end of the incubation period, cell apoptosis was detected by TUNEL-positive staining. (A) Dark brown staining indicted apoptotic nuclei. Arrows indicate the typical features of apoptotic myocytes. (B) More than 200 nuclei from randomly selected fields (10 fields per dish) were counted to calculate the apoptosis index. Data are expressed as the mean ± SEM values from three independent experiments. ^##^P<0.01 compared with vehicle. *P<0.05 compared with ALD. **P<0.01 compared with ALD.

The further confirmation of anti-apoptotic effect of OMT was exhibited via TUNEL assay. The representative morphological views of the vehicle and ALD-treated groups were shown in Figure 3(A). The TUNEL positive cells account for 12.09% in the OMT group and 9.82% in the Spiro group, compared to 15.03% in the ALD group (P<0.05) (Figure 3(B)).

### Relative Signal Molecular Expression of OMT Protected Against ALD-induced Cardiomyocyte Apoptosis

**Figure 4:**
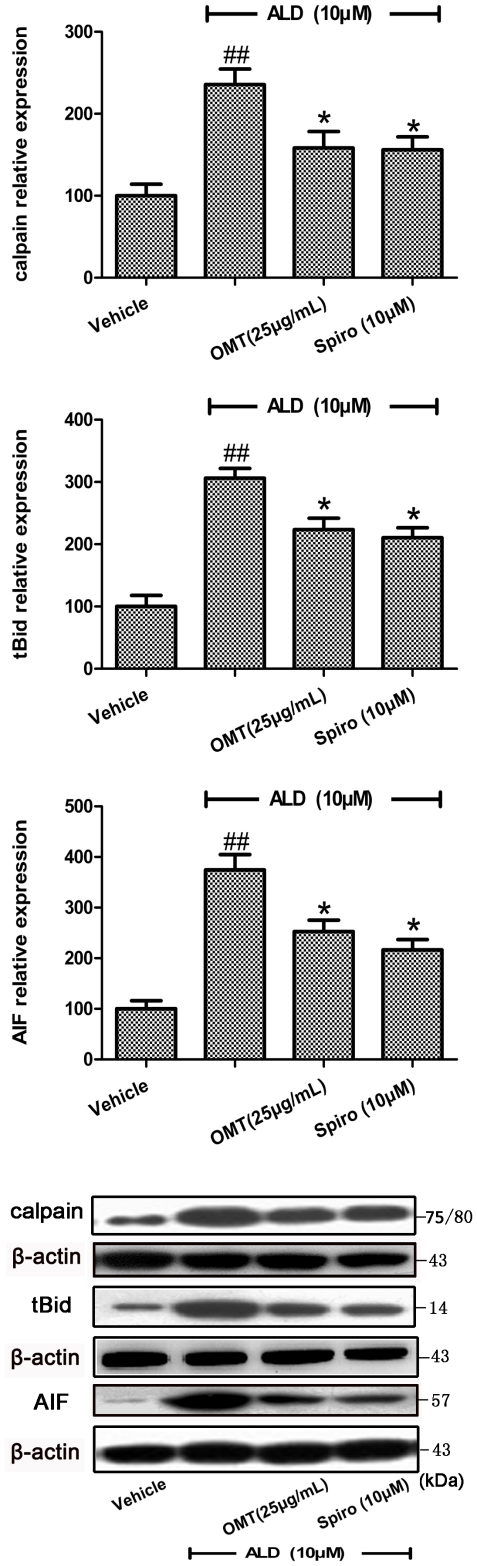
Regulation of OMT on ALD-induced calpain, tBid and AIF levels in cardiomyocytes, respectively. Cardiomyocytes were pre-treated with either Spiro (10 µM), or OMT (25 µg/mL) for 1 h and then were stimulated with ALD (10 µM) for 24 h. At the end of the incubation period, each protein level was analyzed by western blotting. Levels of protein are shown as a percentage of change in the mean value derived from 4 independent experiments. ^##^P<0.01 compared with vehicle. *P<0.05 compared with ALD.

Calpain, cysteine protease, which is activated by sustained elevation of intracellular Ca^2+^, was confirmed to be involved in ALD-induced cardiomyocytes apoptosis. At present, the expression of calpain decreased to (158.33±39.99)% in the OMT group, and also decreased to (156.17±31.25)% in the Spiro group, compared with that in ALD group (235.67±38.03)% (P<0.05) (Figure 4).

Bid, a pro-apoptotic BH3-only member of the Bcl-2 family, is a classic substrate for calpain. The active truncated form, tBid, can promote release of cytochrome C. The present data showed that OMT could inhibit the transformation of Bid induced by ALD, and the level of tBid was (223.54±36.26)% in the OMT group, (210.59±31.91)% in the Spiro group, which were significantly less than that in the ALD group (306.15±31.49)% (P<0.05) (Figure 4).

AIF is thought to promote cell death by binding to DNA and inducing large scale fragmentation, and by increasing mitochondrial permeability. After administration with either OMT or Spiro, the AIF expression in cytosol decreased to (252.73±45.21)% and 216.25±40.84)%, respectively, compared with the ALD group (374.38±60.52)% (P<0.05) (Figure 4).

## Discussion

Evidences from present study indicated that ALD-mediated primary cultured cardiomyocytes damage could be significantly ameliorated by OMT. Meanwhile, the results showed that Spiro could attenuate significantly cardiomyocytes apoptosis due to ALD stimulation. Our previous studies suggested that two different substrates of calcium, calpain- and AIF-mediated pro-apoptotic pathways were involved in the ALD-induced apoptosis [10]. In the present study, the over-expression of pro-apoptotic proteins, including calpain, tBid (calpain target), and AIF, were significantly inhibited because of OMT intervention, and the actions were similar to Spiro.

The cardiomyocytes are terminally differentiated muscle cells with no longer proliferation. Therefore, cardiomyocytes loss with the absence of regenerative capacity may cause irreversible cardiac dysfunction [22], such as heart failure. It is well known that the genesis and development of heart failure are characterized by a number of neurohormonal abnormalities [23], [24]. These include activation of the renin-angiotensin-ALD system (RAAS), which could increase plasma levels of ALD. Besides, ALD can be synthesized and excreted locally in the cardiac tissue, and the ALD system will be activated in heart failure entirely pathological progress. In addition to angiotensin II, elevated potassium concentration, and corticotrophin, etc., the supposed to be short-lived corticotrophin can gradually increase in plasma of patients with heart failure resulting in increase of ALD secretion. The dysregulation of circulating catecholamines, endothelins, and arginine vaspressin in patients with heart failure are also contributing to the high level of ALD in plasma. Moreover, decreased metabolic clearance of ALD by the reduced hepatic perfusion in patients with HF accounts for a several fold increase plasma concentration of ALD in patient with HF [25]. All together, these abnormal increased ALD could produce lots of serious adverse effects on cardiac tissue including cardiac hypertrophy, apoptosis, necrosis and fibrosis, etc.

The [Ca^2+^]_i._ overload is regarded as a pivotal point for lots of signaling pathways leading to pathological status. The role of calcium in ALD signaling-mediated heart failure is a field that has recently been highlighted. For example, the production of reactive oxygen species (ROS) caused by [Ca^2+^]_i._ overload, as a by-product of ALD metabolism, is considered to be one of mechanisms related to ALD-induced cardiotoxicity [26], [27], [28]. In our study, we established the *in vitro* cardiomyocytes injury model by ALD stimulation to induce calcium-related adverse events, which was confirmed by our previous study and other’s observation [3], [9], [10]. ALD co-incubation promoted LDH leakage and decreased the viability of cardiomyocytes, whereas pre-treatment with OMT significantly alleviated the LDH leakage and preserved the viability of cardiomyocytes. These results indicated that OMT could prevent cardiomyocytes from ALD-induced cytotoxity. Moreover, the annexin V/PI and TUNEL staining, and the activity of caspase-3-based evidences suggested that OMT could significantly decrease the apoptosis. Our previous study indicated that ALD-induced cardiomycocytes apoptosis might be mediated by two independent pathways, calpain- and AIF-mediated pro-apoptotic pathways. It is well accepted that over-activated calpains, calcium-dependent cysteine proteases, can result in mitochondrial dysfunction and lead to cell apoptosis [29], [30], [31], [32]. Bid, as calpain target, mediates reactions of mitochondrial proteins, consequently leading to cell death both in *in vivo* and *in vitro* models [33], [34], [35]. Cleavage of Bid results in formation of the active truncated form, tBid, which could promote the release of cytochrome C from mitochondria to cytosol. Several pathways have been proposed for the action of tBid, which may contribute to cytochrome C release followed by caspase-3 activation [36], [37], [38], [39]. AIF, a flavoprotein with NADH oxidase activity anchored to the mitochondrial inner membrane, is known to be relevant to complex I maintenance. During apoptosis, AIF can be released from mitochondria to the cytosol, then to the nucleus, in where it participates in chromatin condensation and large-scale DNA fragmentation. Recent studies on the investigation of the impacts of over-loaded [Ca^2+^]_i._ on cell apoptosis indicated that the translocation of AIF plays indispensable role involved in this process [32]. The down-regulated expression of AIF in cytosol by OMT might be a reason, resulting in less AIF translocation into the nucleus, subsequently, less apoptosis. In the present study, there is a limitation that whether OMT attenuated apoptosis via inhibiting the release of AIF from mitochondria or decreasing the expression of AIF is unclear, which needs to be clarified in the further research.

ALD is present and active all along with the cardiovascular continuum. Excessive ALD signaling results in a multitude of adverse effects on the cardiovascular system. In recent years, with new sites of MR expression being discovered in non-epithelial tissue such as heart, vasculature, brain, etc., the potential ALD target genes in these tissues with unexpected biological functions catch increasing attentions. Besides, there are accumulating evidences from *in vivo* and *in vitro* studies on cross-talk among MR and other molecular signaling pathways, such as MAPK, src, and STAT pathway [40], [41], [42]. So MR blockade will offer tremendous therapy strategies for ALD receptor excessive activation-related diseases. Although the underlying mechanism has not been fully elucidated, at least our studies could provide some clues for the continuing research understand the MR-mediated signaling during cardiac abnormalities. As compared with Spiro, OMT attenuated the cardiac apoptosis, sharing the same way of inhibiting calpain- and AIF-signaling activated in ALD-mediated event. To date, the experimental phenomenon could be explained based on two hypotheses, 1) Sprio and OMT both might intervene with the upstream of the intercellular calcium network pathway; 2) OMT might inhibit ALD-related adverse events via intervening with synthesis of MR, which need to be tested in the further studies. In clinic, there are a variety of pharmacological antagonists on offer, however, they have different side effect profiles which may influence the selection of one drug in preference to the other, and most of them share hyperkalemia as a serious side effect. To find out more effective and safer pharmaceuticals to blockade this neurohormone still claims our intensive attention.

## Conclusions

The present study for the first time demonstrates that OMT could protect cardiomyocytes against ALD-mediated injury, and the beneficial effect was not inferior to Spiro. The mechanism probably related to suppressing the calcium-related calpain and AIF signalings. OMT as a promising potent drug needs further basic and clinical observations in the coming years.

## References

[1] Leal J, Luengo-Fernandez R, Gray A (2012) European Cardiovascular Disease Statistics 2012. 2012 ed. Oxford: The European Heart Network.

[2] Dooley R, Harvey BJ, Thomas W (2011) The regulation of cell growth and survival by aldosterone. Front Biosci 16: 440–457. 10.2741/3697 21196180

[3] Mano A, Tatsum T, Shiraishi J, Keira N, Nomura T, et al (2004) Aldosterone Directly Induces Myocyte Apoptosis Through Calcineurin-Dependent Pathways. Circulation 110: 317–323. 15249508 10.1161/01.CIR.0000135599.33787.CA

[4] Velez Rueda JO, Palomeque J, Mattiazzi A (2012) Early apoptosis in different models of cardiac hypertrophy induced by high renin-angiotensin system activity involves CaMKII. Journal of applied physiology 112: 2110–2120. 22492934 10.1152/japplphysiol.01383.2011 PMC3774203

[5] Ferron L, Ruchon Y, Renaud JF, Capuano V (2011) T-type Ca(2)+ signalling regulates aldosterone-induced CREB activation and cell death through PP2A activation in neonatal cardiomyocytes. Cardiovascular research 90: 105–112. 21123217 10.1093/cvr/cvq379 PMC3058735

[6] Shahbaz AU, Kamalov G, Zhao W, Zhao T, Johnson PL, et al (2011) Mitochondria-targeted cardioprotection in aldosteronism. Journal of cardiovascular pharmacology 57: 37–43. 20966765 10.1097/FJC.0b013e3181fe1250 PMC3022960

[7] Husi H, Sanchez-Nino MD, Delles C, Mullen W, Vlahou A, et al (2013) A combinatorial approach of Proteomics and Systems Biology in unravelling the mechanisms of acute kidney injury (AKI): involvement of NMDA receptor GRIN1 in murine AKI. BMC systems biology 7: 110. 24172336 10.1186/1752-0509-7-110 PMC3827826

[8] Yogi A, Callera GE, O’Connor S, Antunes TT, Valinsky W, et al (2013) Aldosterone signaling through transient receptor potential melastatin 7 cation channel (TRPM7) and its alpha-kinase domain. Cellular signalling 25: 2163–2175. 23838006 10.1016/j.cellsig.2013.07.002 PMC4293696

[9] Zhao J, Li J, Li W, Li Y, Shan H, et al (2010) Effects of spironolactone on atrial structural remodelling in a canine model of atrial fibrillation produced by prolonged atrial pacing. British journal of pharmacology 159: 1584–1594. 20082611 10.1111/j.1476-5381.2009.00551.x PMC2925482

[10] Xiao T, Zhang Y, Wang Y, Xu Y, Yu Z, et al (2013) Activation of an apoptotic signal transduction pathway involved in the upregulation of calpain and apoptosis-inducing factor in aldosterone-induced primary cultured cardiomyocytes. Food and chemical toxicology 53: 364–370. 23266505 10.1016/j.fct.2012.12.022

[11] Mihailidou AS, Loan Le TY, Mardini M, Funder JW (2009) Glucocorticoids activate cardiac mineralocorticoid receptors during experimental myocardial infarction. Hypertension 54: 1306–1312. 19841288 10.1161/HYPERTENSIONAHA.109.136242

[12] Burniston JG, Saini A, Tan LB, Goldspink DF (2005) Aldosterone induces myocyte apoptosis in the heart and skeletal muscles of rats *in* vivo. Journal of molecular and cellular cardiology 39: 395–399. 15907929 10.1016/j.yjmcc.2005.04.001

[13] Soberman J, Chafin CC, Weber KT (2002) Aldosterone antagonists in congestive heart failure. Current opinion in investigational drugs 3: 1024–1028. 12186262

[14] Armstrong PW (2011) Aldosterone antagonists–last man standing? The New England journal of medicine 364: 79–80. 21073364 10.1056/NEJMe1012547

[15] McMurray JJ, O’Meara E (2004) Treatment of heart failure with spironolactone–trial and tribulations. The New England journal of medicine 351: 526–528. 15295043 10.1056/NEJMp048144

[16] Yang YP, Shen XC (2009) Oxymatrine pharmacological action research progress. Chin Hosp Pharm J 29: 405–407.

[17] Cao YG, Jing S, Li L, Gao JQ, Shen ZY, et al (2010) Antiarrhythmic effects and ionic mechanisms of oxymatrine from Sophora flavescens. Phytother Res 24: 1844–1849. 20564505 10.1002/ptr.3206

[18] Gan RT, Dong G, Yu JB, Wang X, Yang SS (2011) Oxymatrine, the Main Alkaloid Component of Sophora Roots, Protects Heart against Arrhythmias in Rats. Planta Med 77: 226–230. 20717872 10.1055/s-0030-1250256

[19] Shen XC, Yang YP, Xiao TT, Peng J, Liu XD (2011) Protective effect of oxymatrine on myocardial fibrosis induced by acute myocardial infarction in rats involved in TGF-beta(1)-Smads signal pathway. J Asian Nat Prod Res 13: 215–224. 21409682 10.1080/10286020.2010.550883

[20] Sun HL, Li L, Shang L, Zhao D, Dong DL, et al (2008) Cardioprotective effects and underlying mechanisms of oxymatrine against Ischemic myocardial injuries of rats. Phytother Res 22: 985–989. 18389484 10.1002/ptr.2452

[21] Zhao J, Yu S, Tong L, Zhang F, Jiang X, et al (2008) Oxymatrine attenuates intestinal ischemia/reperfusion injury in rats. Surg Today 38: 931–937. 18820869 10.1007/s00595-008-3785-8

[22] Tokarska-Schlattner M, Zaugg M, Zuppinger C, Wallimann T, Schlattner U (2006) New insights into doxorubicin-induced cardiotoxicity: the critical role of cellular energetics. J Mol Cell Cardiol 41: 389–405. 16879835 10.1016/j.yjmcc.2006.06.009

[23] Alves AJ, Eynon N, Oliveira J, Goldhammer E (2010) RAAS and adrenergic genes in heart failure: Function, predisposition and survival implications. World J Cardiol 2: 187–197. 21160750 10.4330/wjc.v2.i7.187 PMC2998917

[24] Vijayaraghavan K, Deedwania P (2011) Renin-angiotensin-aldosterone blockade for cardiovascular disease prevention. Cardiol Clin 29: 137–156. 21257105 10.1016/j.ccl.2010.11.003

[25] Weber KT (2001) Aldosterone in congestive heart failure. The New England journal of medicine 345: 1689–1697. 11759649 10.1056/NEJMra000050

[26] Hayashi H, Kobara M, Abe M, Tanaka N, Gouda E, et al (2008) Aldosterone nongenomically produces NADPH oxidase-dependent reactive oxygen species and induces myocyte apoptosis. Hypertens Res 31: 363–375. 18360057 10.1291/hypres.31.363

[27] Manrique C, Lastra G, Gardner M, Sowers JR (2009) The renin angiotensin aldosterone system in hypertension: roles of insulin resistance and oxidative stress. Med Clin North Am 93: 569–582. 19427492 10.1016/j.mcna.2009.02.014 PMC2828938

[28] Manrique C, Lastra G, Habibi J, Wei Y, Morris EM, et al (2007) Methods in the evaluation of cardiovascular renin angiotensin aldosterone activation and oxidative stress. Methods Mol Med 139: 163–179. 18287671 10.1007/978-1-59745-571-8_10

[29] Ajiro K, Bortner CD, Westmoreland J, Cidlowski JA (2008) An endogenous calcium-dependent, caspase-independent intranuclear degradation pathway in thymocyte nuclei: antagonism by physiological concentrations of K(+) ions. Exp Cell Res 314: 1237–1249. 18294629 10.1016/j.yexcr.2007.12.028 PMC2692666

[30] Movsesyan VA, Stoica BA, Yakovlev AG, Knoblach SM, Lea PMt, et al (2004) Anandamide-induced cell death in primary neuronal cultures: role of calpain and caspase pathways. Cell Death Differ 11: 1121–1132. 15375383 10.1038/sj.cdd.4401442

[31] Rizzuto R, Pinton P, Ferrari D, Chami M, Szabadkai G, et al (2003) Calcium and apoptosis: facts and hypotheses. Oncogene 22: 8619–8627. 14634623 10.1038/sj.onc.1207105

[32] Vindis C, Elbaz M, Escargueil-Blanc I, Augé N, Heniquez A, et al (2005) Two Distinct Calcium-Dependent Mitochondrial Pathways Are Involved in Oxidized LDL–Induced Apoptosis. Arterioscler Thromb Vasc Biol 25: 639–645. 15618541 10.1161/01.ATV.0000154359.60886.33

[33] Lee WK, Abouhamed M, Thevenod F (2006) Caspase-dependent and -independent pathways for cadmium-induced apoptosis in cultured kidney proximal tubule cells. Am J Physiol Renal Physiol 291: F823–832. 16597613 10.1152/ajprenal.00276.2005

[34] Mandic A, Viktorsson K, Strandberg L, Heiden T, Hansson J, et al (2002) Calpain-mediated Bid cleavage and calpain-independent Bak modulation: two separate pathways in cisplatin-induced apoptosis. Mol Cell Biol 22: 3003–3013. 11940658 10.1128/MCB.22.9.3003-3013.2002 PMC133754

[35] Zhang YM, Bhavnani BR (2006) Glutamate-induced apoptosis in neuronal cells is mediated via caspase-dependent and independent mechanisms involving calpain and caspase-3 proteases as well as apoptosis inducing factor (AIF) and this process is inhibited by equine estrogens. BMC NEUROSCI 7: 49–71. 16776830 10.1186/1471-2202-7-49 PMC1526740

[36] Henry-Mowatt J, Dive C, Martinou JC, James D (2004) Role of mitochondrial membrane permeabilization in apoptosis and cancer. Oncogene 23: 2850–2860. 15077148 10.1038/sj.onc.1207534

[37] Kroemer G, Reed JC (2000) Mitochondrial control of cell death. Nat Med 6: 513–519. 10802706 10.1038/74994

[38] Orrenius S, Zhivotovsky B, Nicotera P (2003) Regulation of cell death: the calcium-apoptosis link. Nat Rev Mol Cell Biol 4: 552–565. 12838338 10.1038/nrm1150

[39] Zhao Y, Ding WX, Qian T, Watkins S, Lemasters JJ, et al (2003) Bid activates multiple mitochondrial apoptotic mechanisms in primary hepatocytes after death receptor engagement. Gastroenterology 125: 854–867. 12949730 10.1016/s0016-5085(03)01066-7

[40] Callera GE, Yogi A, Briones AM, Montezano AC, He Y, et al (2011) Vascular proinflammatory responses by aldosterone are mediated via c-Src trafficking to cholesterol-rich microdomains: role of PDGFR. Cardiovasc Res 91: 720–731. 21576132 10.1093/cvr/cvr131

[41] Huang LL, Nikolic-Paterson DJ, Ma FY, Tesch GH (2012) Aldosterone Induces Kidney Fibroblast Proliferation via Activation of Growth Factor Receptors and PI3K/MAPK Signalling. Nephron Exp Nephrol 120: e114–e121. 10.1159/000339500 22814207

[42] Tirard M, Jasbinsek J, Almeida OF, Michaelidis TM (2004) The manifold actions of the protein inhibitor of activated STAT proteins on the transcriptional activity of mineralocorticoid and glucocorticoid receptors in neural cells. J Mol Endocrinol 32: 825–841. 15171715 10.1677/jme.0.0320825

